# Assessing the Policy Landscape for Salt Reduction in South-East Asian and Latin American Countries – An Initiative Towards Developing an Easily Accessible, Integrated, Searchable Online Repository

**DOI:** 10.5334/gh.929

**Published:** 2021-07-15

**Authors:** Aprajita Kaushik, Frank Peralta-Alvarez, Priti Gupta, Juan Carlos Bazo-Alvarez, Sandra Ofori, Kirsty Bobrow, Dan Monyeki, Renzo R. Guinto, Jill Baumgartner, Sailesh Mohan

**Affiliations:** 1 Centre for Chronic Disease Control, IN; 2 Universidad Católica Sedes Sapientiae, PE; 3 Research Department of Primary Care and Population Health, University College London, London, UK; 4 Escuela de Medicina, Universidad Cesar Vallejo, Trujillo, Peru; 5 University of Port Harcourt, NG; 6 Department of Medicine, University of Cape Town, ZA; 7 University of Limpopo, Sovenga, ZA; 8 St. Luke’s Medical Center College of Medicine, Quezon City, PH; 9 McGill University, CA; 10 Public Health Foundation of India, IN; 11 Deakin University, AU

**Keywords:** salt, salt reduction, salt policy, CVD, South-East Asia, Latin America

## Abstract

**Background:**

High dietary salt intake is an avoidable cause of hypertension and associated cardiovascular diseases (CVDs). Thus, salt reduction is recommended as one of the most cost-effective interventions for CVD prevention and for achieving the World Health Organization’s (WHO) 25% reduction in premature non-communicable disease (NCD) mortality by 2025. However, current and comprehensive information about national salt reduction policies and related actions across different regions are difficult to access and impede progress and monitoring.

**Objectives:**

As an initial step to developing an online repository of salt reduction policies and related actions, and to track nation-wise progress towards the WHO’s 25 by 25 goal, we aimed to identify and assess salt reduction policies and actions in select countries from two of the top five most populous regions of the world- the South-East Asia and Latin America.

**Methods:**

We conducted a literature review to identify national and regional salt reduction policies in the selected South-East Asian and Latin American countries, from January 1990–August 2020, available in English and Spanish. We also contacted selected WHO country offices (South-East Asian region) or relevant national authorities (Latin America) to gain access to unpublished documents.

**Results:**

In both regions, we found only a few dedicated stand-alone salt reduction policies: Bhutan, Sri-Lanka and Thailand from South East Asia and Costa Rica from Latin America. Available polices were either embedded in other national health/nutritional policy documents/overall NCD policies or were unpublished and had to be accessed via personal communication.

**Conclusions:**

Salt reduction policies are limited and often embedded with other policies which may impede their implementation and utility for tracking national and international progress towards the global salt reduction target associated with the 25 by 25 goal. Developing an online repository could help countries address this gap and assist researchers/policymakers to monitor national progress towards achieving the salt reduction target.

## Introduction

The burden of noncommunicable diseases (NCDs) globally is considerably high. According to the World Health Organization (WHO), in 2016, 71% (i.e., 41 million) of the 57 million global deaths were attributable to NCDs. Of these, cardiovascular diseases (CVDs) were the number one cause of deaths globally, accountable for 31% of all global deaths and 44% (17.9 million) of all NCD related deaths [1]. In 2011, the UN General Assembly adopted a political declaration that committed its member countries to adopt a global monitoring framework comprising of nine voluntary global targets. These targets aimed at a 25% reduction in premature mortality from NCDs by 2025 (25 × 25 goals) [2].

Reducing the NCD risk factors and meeting the 25 × 25 goals can be achieved through cost-effective national and regional public health policies. The WHO recommends a package of evidence-based interventions and policy options called the ‘Best Buys’ for countries to achieve the proposed NCD targets [3]. One of these ‘Best Buys’ includes policies on addressing salt reduction as high blood pressure — a major risk factor for CVDs, is caused due to high dietary salt intake [4]. Lowering the dietary salt intake among the general population has been an important determinant of the decrease in CVD mortality in some high-income countries, although locally applicable salt reduction strategies are still required in low- and middle-income countries, where salt intake remains high [5,6]. WHO recommends reducing salt intake to less than 5 g/d per person (approximately equivalent to a teaspoon) [7].

Currently, there is a need to assess the policy landscape for salt reduction across countries, in order to be able to track their progress towards the 25 × 25 goals. To enable this comparison of salt reduction policies, we aimed to begin with identifying existing salt reduction policies in the two of the top five most populous regions of the world- South-East Asia and Latin America. According to the WHO country profiles, all the selected countries from these regions had a mean population salt intake above the WHO recommended 5 g/d, while data from various studies as well as from the Global Burden of Disease study (GBD) has highlighted the increasing burden of CVDs in these regions [8,9] (see Tables 1 and 2). In Latin America, CVDs accounted for 31% of all deaths, while across the South-East Asian countries, of the 65% deaths occurring due to NCDs, 18% to 40% of these were attributable to CVDs [1]. As of 2016, 78% of all NCD related deaths and over 75% of CVD deaths occurred in low- and middle-income countries (LMICs) such as those in the aforementioned regions, which were the highest, as compared to those in high-income countries (HICs).

**Table 1:** Summary of overall CVD mortality, CVD mortality due to diets high in sodium, mean salt intakes (g/d) and existing salt reduction policies and actions in the selected South-East Asian countries.

Country	Overall CVD mortality	CVD mortality due to diets high in sodium	Mean salt consumption (g/d)	Salt reduction policy	Actions

**Bangladesh**	298.0 (269.6–326.2)	36.4 (6.6–75.1)	9.0	Unavailable	Salt reduction campaigns. Consumer awareness through mass media and social media.
**Bhutan**	217.1 (182.5–255.0)	29.9 (6.3–60.3)	9.0	National Salt Reduction Strategy (2018–2023)	Strengthening regulatory measures. Increasing knowledge and awareness. Promoting healthy settings. Strengthening evidence generation, monitoring and evaluation.
**India**	282.3 (265.0–293.3)	16.5 (1.7–44.6)	9.0	NCD Multisectoral Action Plan (2017–2022)	Legislation developed on nutrient content panel that includes salt. Policy on front of pack food labelling drafting in process. Voluntary pledges by industry to reduce salt, sugar and fat.
**Indonesia**	342.9 (324.4–364.7)	69.8 (28.0–116.2)	9.0	National Action Plan on NCDs 2015–19 and 2020–2024	Developed and adapted national policies on population salt reduction. Conducting a total dietary survey and RISKESDAS (a national household survey) for verifying achievement of 10% salt reduction by 2019 target; i.e., a 10% reduction in salt intake.
**Maldives**	164.9 (154.3–175.6)	32.7 (13.8–54.7)	8.0	Multisectoral Action Plan for Prevention & Control of Non-communicable 2016–2020	Improving consumer knowledge and awareness. Conducting a pilot study on salt consumption.
**Nepal**	260.8 (227.6–292.3)	29.1 (4.2–63.3)	10.0	Multisectoral Action Plan for the Prevention and Control of Non-Communicable Diseases (2014–2020)	Carrying out a STEPS survey to assess age-standardized mean population intake of salt/sodium (gm/day) among those aged 18+years.
**Sri-Lanka**	197.1 (171.6–220.2)	36.4 (13.4–62.0)	10.0	National Salt reduction strategy (2018–2020)	Developing regulations. Obligating the food industry to promote food reformulation. Implementing a colour-coded labelling strategy (includes front of pack labelling and traffic light labelling to be implemented by 2019). Enforcing salt-reduction laws. Enhancing consumer knowledge and awareness (a sodium reduction behavior changes under development).
**Thailand**	109.9 (100.5–121.5)	19.1 (7.2–32.7)	13.0	Salt and Sodium reduction Policy (2016–2025)	Labelling and legislation (include mandatory nutrient content labelling). Product reformulation. Consumer knowledge and awareness. Developing a nation-wide salt reduction campaign. Reforming environment by promoting availability of low salt products and food options. Developing monitoring tools and evaluating data on salt consumption trends.

**Table 2:** Summary of overall CVD mortality, CVD mortality due to diets high in sodium, mean salt intakes (g/d) and existing salt reduction policies and actions in the selected Latin American countries.

Country	Overall CVD mortality	CVD mortality due to diets high in sodium	Mean salt consumption (g/d)	Salt reduction policy	Actions

Argentina	191.0 (174.9–209.0)	16.0 (1.2–38.3)	8.0	Unavailable	Survey determining an acceptable concentration of salt for consumers. Agreements with bakery industries to reduce the amount of sodium in processed foods. Conducting local and national evaluation to monitor sodium content.
Brazil	178.0 (175.9–180.0)	20.0 (3.7–41.1)	10.0	Unavailable	Reduction of sodium amount in processed foods. Educational sessions for increasing population’s awareness. Developing nutritional practice guidelines.
Chile	128.0 (117.0–139.5)	18.1 (3.7–35.3)	7.0	Unavailable	Educational awareness campaigns for decreasing added salt in processed foods. Efforts towards decreasing the concentration of salt in bread across nation.
Colombia	124.2 (113.7–135.4)	14.7 (2.9–29.9)	10.0	Unavailable	Voluntary agreements with the food industry and research on communication. Designing the protocol to determine the baseline sodium intake.
Peru	85.8 (75.7–96.1)	12.7 (0.9–29.6)	8.0	Ley de la Alimentación Saludable: Manual de Advertencias Publicitarias (2018)	Labeling products containing high salt.
Uruguay	160.7 (147.3–174.8)	9.0 (0.1–27.7)	7.0	Unavailable	Banning table salt and salty condiments from restaurants. Implementing low salt options in restaurants.
Costa Rica	138.0 (130.1–146.5)	7.2 (0.1–22.4)	8.0	The National Plan to Reduce Public Consumption of Salt/Sodium in Costa Rica (2011–2021)	Determining sodium intake and salt/sodium content of widely consumed foods. Identifying consumer knowledge, attitudes, and behaviours regarding salt/sodium intake, and nutritional labeling. Promoting behavioural changes among the general population to reduce dietary salt intake. Implementing strategies to reduce the salt/sodium content of processed foods and foods prepared at home. Monitoring and evaluation of salt/sodium intake in population.
Cuba	191.0 (174.1–208.8)	8.9 (1.2–19.6)	7.0	National Program of Non-Communicable Diseases (2010)	Mass media campaigns. Nutrition labelling (including salt). Workshops on dietary salt reduction. National implementation of Cuban dietary guidelines. Conducting studies on knowledge attitudes and beliefs. Coordinating salt reduction program with the iodine supplementation program.
Mexico	152.8 (149.8–156.2)	20.4 (4.0–38.9)	7.0	National Agreement for Nutritional Health—Strategy to Control Overweight and Obesity (2010)	Limiting the amount of sodium added to foods. Reducing dietary sodium intake.

## Materials and Methods

### Search Strategy

A systematic search strategy was used to identify national and regional policies on salt reduction in select South-East Asian and Latin American countries. Our selected countries included Bangladesh, Bhutan, India, Indonesia, Maldives, Nepal, Sri-Lanka, Thailand, Argentina, Brazil, Chile, Colombia, Costa-Rica, Cuba, Mexico, Peru and Uruguay. We conducted a systematic search for peer reviewed literature on Pub-med database and on Google Scholar to identify for relevant policy documents mentioning ‘salt reduction’ in English and Spanish from January 1990 to August 2020.

Articles were searched on Pub-med by using the terms ‘salt’, ‘policy’, ‘sodium’, ‘salt reduction’, ‘salt intake’, ‘salt policy’, ‘salt policies’, ‘salt law’, ‘South East Asia’, ‘Bangladesh’, ‘Bhutan’, ‘India’, ‘Indonesia’, ‘Maldives’, ‘Nepal’, ‘Sri Lanka’, ‘Thailand’, ‘Argentina’, ‘Brazil’, ‘Chile’, ‘Colombia’, ‘Costa Rica’, ‘Cuba’, ‘Mexico’, ‘Peru’ and ‘Uruguay’. Google scholar was searched for relevant and existing grey literature related to salt reduction policies in these countries. In parallel, searches for relevant salt reduction policy documents on WHO country websites, National Ministry of Health (MOH) websites, National Institute of Nutrition (NIN), Indian Council of Medical and Research (ICMR), World Heart Foundation (WHF) and The Pan-American Health Organization (PAHO) websites were carried out. This search was further supplemented with information on CVD mortality from the Institute of Health Metrics and Evaluation (IHME) website while WHO country profiles were used to access information on national mean salt intakes (g/d).

A request was sent to the WHO country offices through the South-East Asia Regional (SEAR) office in order to obtain information on the status of salt reduction policies and efforts. It included country-wise details of the information we had accessed through our search, followed by a question asking for updates on the current situation in the country pertaining to salt reduction. WHO representatives from Bhutan, Nepal, Sri-Lanka and Thailand were contacted personally via e-mails. For Latin American countries, a search for authorities related to nutrition policy development was conducted. For each country, a leader was identified and then contacted by email in order to obtain information about salt reduction initiatives.

### Inclusion Criteria

Studies/ documents were included based on the following criteria: 1. Refers to the policies on salt reduction (drafted or implemented), 30% relative reduction in the mean population salt/sodium intake, and any salt reduction efforts (by NGOs, food industries, governments etc.) in the selected South-East Asian and Latin American countries; and 2. Published in English or Spanish from January 1990–August 2020.

### Data Synthesis

Information related to salt reduction policies was extracted for the selected countries in South-East Asia and Latin America. Key components that were assessed included efforts to enhance salt reduction research and evaluation, voluntary efforts such as increased awareness about risks of high salt intake and educative campaigns using mass-media, laws/ policies by the government and efforts taken by food industries to reduce salt content in processed foods. Sodium intake was reported as mg/day and salt intake was reported as g/day. We defined the published policies into two categories: 1. a standalone policy, that is, a dedicated policy on salt reduction that is available as an independent document and 2. an embedded policy, that is, a dedicated policy on salt reduction which is a part of another policy document.

## Results

**Figure 1:**
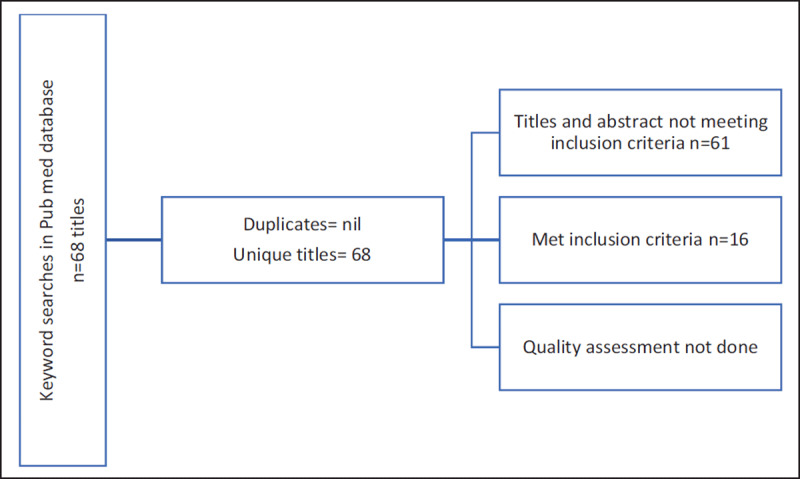
Summary of findings from Pub-med database for selected South-East Asian and Latin American countries.

The search strategy yielded 68 potentially relevant articles on Pub-med. After reviewing the titles and abstracts, 16 articles fulfilled the inclusion criteria, and were included in this narrative synthesis. (See Figure 1 for search summary, see Tables 1 and 2 for existing salt reduction policies and actions in the selected South-East Asian and Latin American countries, respectively).

### South-East Asian Countries

#### Bangladesh

Information from the WHO country office revealed that after having completed the situational analysis, Bangladesh is currently in its early stage of policy development in terms of reducing population salt intake. The National Multisectoral Action Plan for Prevention and Control of Noncommunicable Diseases (2018–2025) provided insights more on the voluntary efforts being taken in the country, such as salt reduction campaigns and conducting public campaigns through mass media and social media to inform consumers than on dedicated stand-alone salt reduction policies [10].

#### Bhutan

The main objectives of the National Salt Reduction Strategy (2018–2023) (access to which was gained via communication with the WHO country office) were increasing information, education and communication, and promoting healthy settings. A national healthy diet strategy is being implemented in the country, which includes school food procurement and sodium reduction. There has been no implementation of any other salt policy measures [11].

#### India

The National Multisectoral Action Plan for Prevention and Control of Common Non-communicable diseases (2017–2022) did not indicate any dedicated stand-alone salt reduction policies in the country or any voluntary efforts being undertaken [12]. Information from the WHO country office revealed that a legislation is being developed on nutrient content panel, which includes salt as well. A policy on front of pack food labelling was drafted and voluntary pledges by the food industry to reduce salt content are also being considered.

#### Indonesia

The National Strategic Action Plan for the Prevention and Control of Non-communicable diseases (2016–2019) indicated that national policies to reduce population salt/sodium consumption were developed and adapted in the country [13]. However, our search could not yield any documentation of these policies. Information from the WHO country office revealed that ministerial regulations on warning labels regarding high salt are yet to be implemented.

#### Maldives

The National Multi-Sectoral Action Plan for the Prevention and Control of Non-communicable diseases in Maldives (2016–2020) focused on indicators that included improving knowledge and awareness about high salt intake, conducting STEPS survey and assessing the mean population intake of salt (g/d) in persons aged 18+ years [14]. There were no specific actions on labelling or settings-based salt reduction.

#### Nepal

The National Multi-Sectoral Action Plan for Prevention and Control of Non-communicable diseases (2014–2020) focused on pilot urinary salt assessment and carrying out a STEPS survey to assess age-standardized mean population intake of salt/sodium (g/d) in 18+years persons, as the key targets [15]. Information from the WHO country office indicated that an action plan is being developed for salt reduction.

#### Sri-Lanka

Access to the executive summary of National Salt-reduction Strategy (2018–2020) was gained from personal communication [16]. The Government of Sri-Lanka launched an initiative ‘National Strategic Plan 2018–2022’ to reduce the population dietary salt intake as a part of the National Multisectoral Action Plan for The Prevention and Control of Noncommunicable Diseases (2016–2020) [17]. Key targets included obligating the food industry to promote food reformulation, deciding standards of labelling, enhancing consumer knowledge and awareness and developing and implementing the National Salt-reduction Strategy. Currently, a front of pack labelling and traffic light labelling approach for salt reduction are being considered for implementation. A behaviour change sodium reduction campaign is also under development.

#### Thailand

According to the WHO country-website, the Ministry of Public Health implemented the Salt and Sodium Reduction Policy (2016–2025), which was accessed from the WHO country office [18]. The action plan focused on labelling, legislation, salt-reduced foods reformulation and prioritizing consumer education. A nutrient content label including salt is mandatory in the country. Sodium taxation is currently under discussion.

#### Selected Latin American countries

A currently active project aims to expand salt reduction programs through research in 5 Latin American countries- Argentina, Brazil, Costa Rica, Paraguay and Peru [19]. The PAHO website has advertised various advocacy materials on salt reduction interventions and indicates steps towards voluntary initiatives for salt-reduction, such as the Salt Awareness Week from 4^th^–10^th^ March each year- that aims to encourage the implementation of evidence-based interventions for salt intake reduction [20].

#### Argentina

Argentina’s approach includes both mandatory targets for packaged food and regulated limits on sodium content of key foods, such as bread products as well as voluntary targets for local producers, such as small bakeries [21]. The food industry complies to a national law that limits salt content of their products. More than 84% of products have achieved a salt level below the maximum permitted level [22]. Another initiative by the government — ‘Less Salt More Life’ aims at reducing salt consumption in the entire population by reducing salt use during cooking or at the table [23].

#### Brazil

By implementing strategies such as nutritional practice guidelines, the country’s government and the food industry settled to establish voluntary, gradual and sustainable targets to reduce the maximum sodium content of industrial foods [24].

#### Chile

Chile adopted a traffic light rating system indicating salt content in foods using the red, amber, or green of traffic lights. The green, amber and red colour refer to foods with low salt (<0.3 g per 100 g), medium salt (0.3 g–1.5 g per 100 g) and high salt (>1.5 g per 100 g), respectively. In 2015, it adopted mandatory warning labels for packaged foods and implemented the printing of octagonal symbol (with the description ‘Alto en Sodio’) for products with high levels of sodium (>800 mg–100 mg) [25,26,27,28].

#### Colombia

Columbia’s food industry opted for voluntary agreements and research strategies for increasing awareness among the general population regarding the risks of high salt intake. Efforts with the School of Nutrition and Dietetics at the University of Antioquia were made to design a protocol for determining the baseline sodium intake [29]. The country is now considering a bill to implement FOP warning labels in a new nutrient labelling system [30].

#### Costa Rica

In Costa Rica, the food industry follows the Central American Technical Regulation for General Labelling of Food Products and the Central American Technical Regulation of Nutrition Labelling. However, they are based on voluntary declaration of critical nutrients such as high sodium and there have been no efforts to implement an evidence-based labelling system [31]. The country has an initiative called The National Plan to Reduce Public Consumption of Salt/Sodium in Costa Rica 2011–2021. Although the country aims to meet the WHO’s salt reduction targets, the efforts are hampered by limited interinstitutional coordination and lack of data around national salt intake levels [32].

#### Cuba

The 2010 National Program of Non-Communicable Diseases aims to reduce salt intake by promoting nutritional labelling (including salt), conducting workshops on reducing dietary salt by coordinating the salt reduction efforts with the iodine supplementation program [33].

#### Mexico

The National Agreement for Nutritional Health—Strategy to Control Overweight and Obesity document, which is the result of collaborative efforts among the Mexican government, public sector and academia, includes strategies for limiting the amount of sodium intake by reducing addition of salt to foods [34]. Approved in 2020, as a part of the General Health Law, Mexico has adopted a front-of-pack labelling strategy which aims at food and beverage manufacturers to include a warning labels in the shape of black octagons on products that are high in salt [35,36].

#### Peru

Peru currently has no salt reduction strategy [37]. However, studies from the country have suggested that salt related interventions are easily implementable and have the potential to contribute to larger salt reduction efforts [38]. In 2018, the document ‘Manual de Advertencias Publicitarias’ was published as a complement of the national policy ‘Ley de la Alimentación Saludable’, according to which, all products with high levels of sodium must be labelled with a black octagon, with the warning ‘ALTO EN SODIO’ or ‘high in sodium’ [39,40].

#### Uruguay

Montevideo, the capital city of Uruguay, has taken efforts to ban table salt and salty condiments, such as ketchup and mayonnaise, from restaurants. Restaurants are also mandated to provide low salt options to their customers and add warnings of high salt content to menus [41]. Similar to Chile and Peru, Uruguay has also adopted nutritional warnings in the form of black octagons indicating ‘high in sodium’ or ‘excessive sodium’ [40].

## Discussion

**Figure 2:**
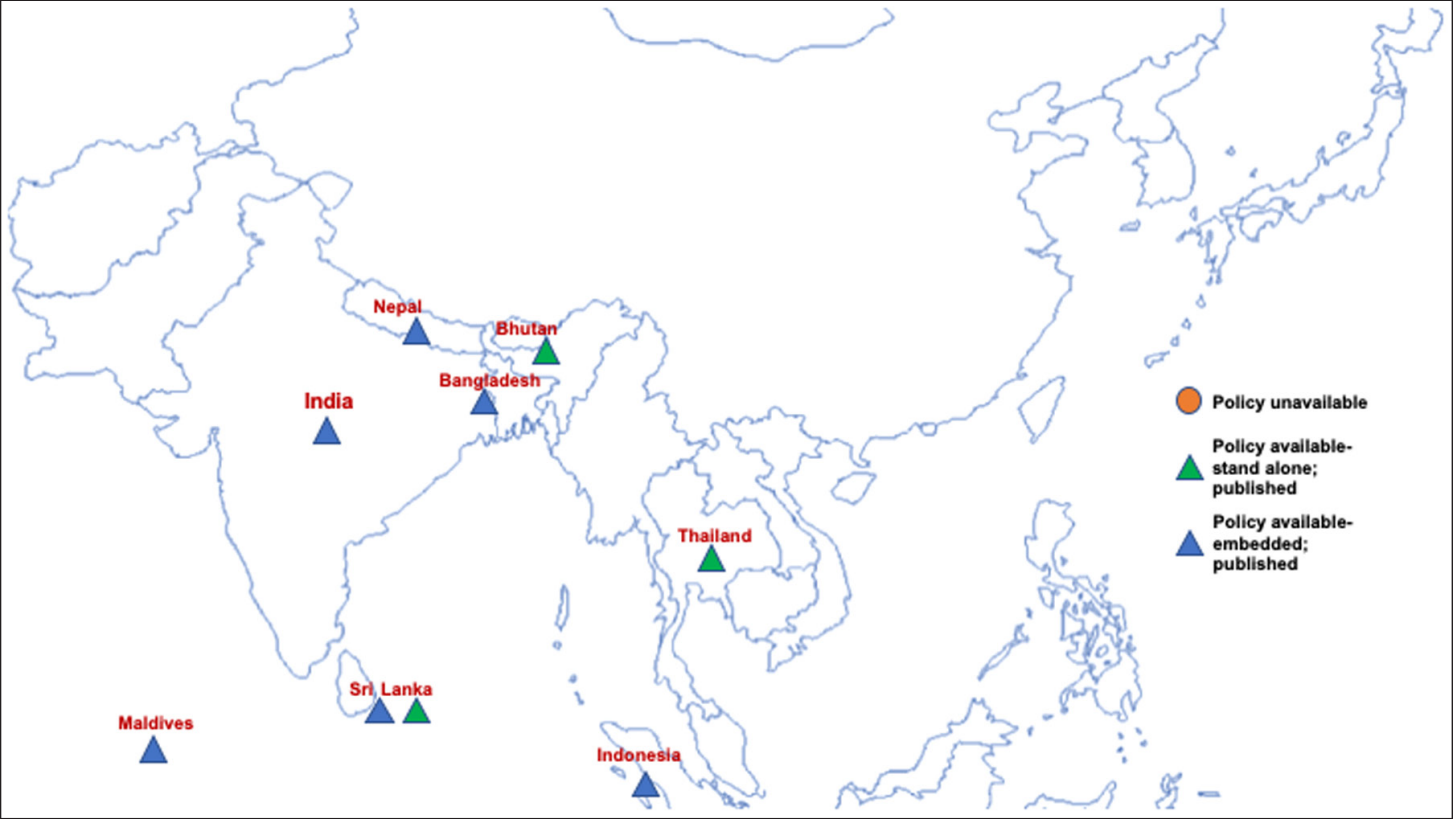
Map of selected South-East Asian countries highlighting the presence of salt reduction policies.

**Figure 3:**
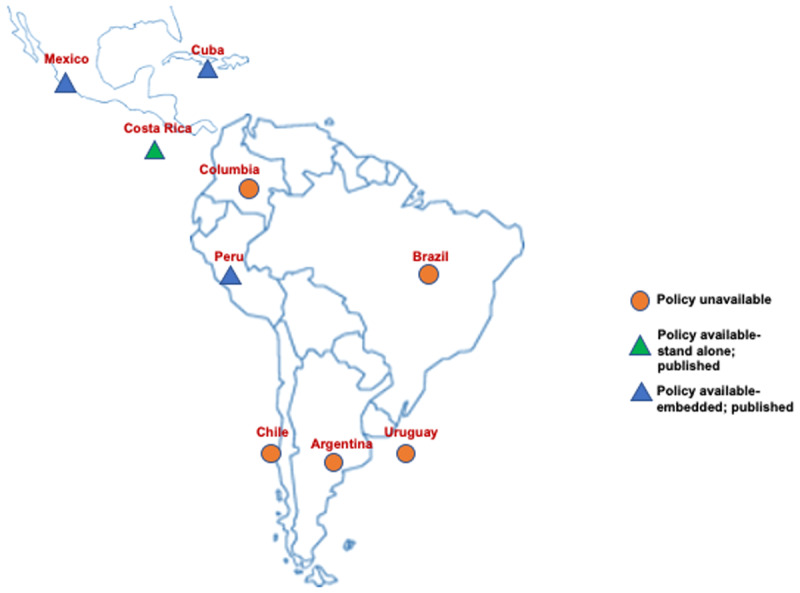
Map of selected Latin American countries highlighting the presence of salt reduction policies.

Despite high levels of CVD mortality and CVD mortality due to high dietary salt intake as well as the global mandate to reduce salt intake to achieve the 25 × 25 goal, our study revealed that there was a lack of dedicated stand-alone salt reduction policies in both South-East Asian as well as Latin American countries, except for Bhutan, Sri Lanka, Thailand (see Figures 2 and 3) and Costa Rica (see Figure 3), respectively. The policies that were available from other selected countries were mostly embedded within National Multisectoral Action Plans, (for example India, Indonesia and Peru) or other policies (for example, The National Agreement for Nutritional Health —Strategy to Control Overweight and Obesity, Mexico).

In South-East Asia, countries like Bangladesh, India, Nepal, Maldives and Indonesia lacked a dedicated policy focusing on salt reduction in the country (Figure 2). For example, according to the NCD Multisectoral Action Plan (2017–22), voluntary pledges by the food industry and a front of food pack labelling policy is in process in India, however focused initiatives towards salt reduction are missing. Similarly, in Latin America, countries like Argentina, Brazil, Columbia, Chile and Uruguay lack a dedicated salt reduction policy, whether embedded or standalone, however, Cuba, Mexico and Peru had published dedicated salt reduction policies although embedded in other National policies/documents.

Our search also revealed that despite the economical and developmental differences across the two selected regions, most countries had limited salt reduction policies. A study assessing the global implementation of WHO recommended NCD policies, revealed a mean 2017 implementation score of 0.4 for salt policies (for a range where 0.5 indicates partial implementation and 1.0 indicates complete implementation) [42]. In South-East Asia, a few countries were moving towards developing policies on salt reduction, although most of the efforts were voluntary and revolved around labelling and educational awareness. Only Sri-Lanka and Thailand had dedicated stand-alone policies for salt reduction and adopted strict regulations obligating food products reformulation and labelling policies, such as implementing a colour-coded labelling strategy and front of pack labelling.

On the other hand, Latin American countries such as Chile and Argentina were doing similarly well in terms of approaching the salt reduction targets. Chile adopted traffic light labelling strategies while Argentina placed mandatory limits on salt content of packaged food, however both the countries lacked a dedicated stand-alone national salt reduction policy – contrary to Sri Lanka and Thailand. Costa Rica had a dedicated policy document –The National Plan to Reduce Public Consumption of Salt/Sodium in Costa Rica, although the country lacked data on national salt intake levels, which made policy implementation arduous.

As per our results, both South-East Asian as well as Latin American countries, despite differences in their socio-economic status and healthcare systems, had a similar status in terms of salt reduction policies. Most countries implemented voluntary targets for salt reduction but lacked dedicated stand-alone salt reduction policies as well as systems for monitoring the progress towards these targets. Keeping in mind the increasing burden of CVDs and hypertension, an important element of which is salt reduction, it is disconcerting to observe lack of implementation of national salt reduction despite it being recommended as a ‘Best Buy’ by WHO.

Therefore, in terms of addressing the 2025 target of 30% mean population salt intake reduction, countries are at different levels with respect to policies and their implementation. There is also a lack of updated data on salt intake among country’s populations alongside lack of monitoring and surveillance. One of the major challenges we faced during our search was gaining access to the policies on salt reduction across all countries. For instance, the Salt and Sodium Reduction Policy (2016–2025), Thailand had to be accessed via personal communication, while Sri Lanka’s National Strategic Plan 2018–2022 was a part of the National Multisectoral Action Plan for The Prevention and Control of Noncommunicable Diseases (2016–2020).

Due to the unavailability of policy related information online and the absence of a single integrated platform for monitoring policy-related progress, it was challenging to assess the country-level status in terms of salt reduction initiatives and to monitor the progress towards achieving the 25 × 25 goals. The absence of an accessible database for policies and monitoring the nation-wide implementation status, makes retrieving information even through a robust search strategy, a major challenge. To access relevant information a lot of personal communication had to be resorted to, which in the long term is an infeasible method for identifying new and existing policies and tracking global as well as national progress.

Therefore, in order to avoid the current scenario of scattered information and the lack of country-wise salt intake information as well as policies, there is an important need to develop a repository that can bring together national salt reduction policies and information about salt reduction initiatives on a single accessible platform. Such a repository will allow different stakeholders such as health policy makers, implementors and researchers easy and timely access for comparisons, monitoring and learning. This study is a first step towards our objective of building an online repository of national and regional policies, which will help researchers and policymakers to track and monitor nation-wise status in terms of salt reduction policy development as well as the degree of implementation for achieving the broader 30% salt reduction target, and the 25 × 25 goals. We will develop and pilot an online format for the repository that will include searchable library of policies and actions in member states and a series of data visualisations that will facilitate commitment and actions. In addition, end-users will be able to incorporate intake data and upload polices directly onto the database. This can later be extended to a surveillance platform which will help in monitoring the impact of salt reduction policies and initiatives on CVD mortality trends. For sustainability, we plan to link the repository to the WHF website with the system that flags up when new policies are added using specific keywords.

## Conclusion

There is a lack of evidence-based dedicated stand-alone salt reduction policies in both South-East Asian and Latin American countries. All the policies that are present, are scattered or embedded with in other documents, making their access and monitoring nation-wise progress difficult. Not only salt reduction strategies, there is a lack of updated data on salt intake, monitoring and surveillance. This situation can likely impede monitoring of the salt reduction efforts and hamper national and global progress towards achieving the global salt reduction target associated with the 25 by 25 goal. Our aim of building an online repository of national and regional policies will be beneficial to both researchers and policymakers by helping them incorporate salt intake data as well as track and monitor nation-wise status in terms of salt reduction policy development and their implementation. This will further facilitate country level progress towards achieving the 2025 global target of 30% salt reduction and addressing the gaps between data collection, monitoring and surveillance, policy development and policy implementation.
